# *Mycobacterium Intracellulare* Infection of the Metacarpophalangeal Joint in a Patient With Rheumatoid Arthritis: A Case Report

**DOI:** 10.1155/crrh/1818066

**Published:** 2025-08-28

**Authors:** Ryosuke Hanaoka

**Affiliations:** Department of Rheumatology and Internal Medicine, Kamitsuga General Hospital, Kanuma, Tochigi, Japan

## Abstract

**Background:** Nontuberculous *Mycobacterium* (NTM) infections affecting musculoskeletal structures are rare, particularly in patients with well-controlled rheumatoid arthritis (RA). This case is reported to highlight the potential risk of focal tenosynovitis due to *Mycobacterium intracellulare* following intra-articular glucocorticoid injection.

**Case presentation:** A 79-year-old man with well-controlled RA developed tenosynovitis with bone destruction in the right index finger metacarpophalangeal joint following a single intra-articular injection of triamcinolone acetonide. Despite antibiotic treatment, the condition progressively worsened. Synovectomy revealed *Mycobacterium intracellulare* infection involving both flexor tendons, joint space, and bone marrow. The patient regularly engaged in gardening activities without protective gloves.

**Conclusion:** This case highlights the importance of considering NTM infection in the differential diagnosis of persistent monoarthritis that worsens after intra-articular glucocorticoid injection, especially in patients with exposure risk factors such as gardening.

## 1. Introduction

Rheumatoid arthritis (RA) is a systemic inflammatory disease of unknown etiology, which causes irreversible articular destruction via an autoimmune mechanism [1]. In recent years, more aggressive treatments aiming at clinical remission by using methotrexate, Janus kinase inhibitors, or various biological agents have been performed in patients with RA [2]. However, it is not usually easy to achieve clinical remission as defined by fulfillment of the Boolean criteria. Moreover, most physicians frequently face difficulty in completely preventing arthritis over a long period of time. Occasional monoarthritis in commonly used joints despite aggressive treatments for RA is not rare. Intra-articular injection of glucocorticoids, such as triamcinolone acetonide, is routinely performed for monoarthritis in patients with well-controlled RA [3]. However, we must be aware of the possibility of the occurrence of infectious arthritis or osteomyelitis as adverse effects of intra-articular injection of glucocorticoids.

Nontuberculous *Mycobacterium* (NTM) are environmental bacterium that naturally occurs in soil and water. About 150 types of nontuberculous *Mycobacteria* have been identified, of which about 30 have been reported to cause human infections. The most frequent of these are *Mycobacterium avium* and *Mycobacterium intracellulare*, which are together called as *Mycobacterium avium–intracellulare* complex (MAC). MAC infects mainly middle-aged and elderly women and causes chronic pulmonary infection. On the other hand, musculoskeletal infections, such as arthritis, tenosynovitis, and osteomyelitis due to MAC infection have only rarely been observed [4].

Here, we report a rare case of infectious osteomyelitis, tenosynovitis, and arthritis caused by *M. intracellulare*, which was associated with a preceding intra-articular triamcinolone acetonide injection.

## 2. Case Presentation

A 79-year-old man was admitted to our hospital presenting with painful swelling of the right index finger. His medical history included RA, diagnosed 7 years ago when he presented with polyarthralgia. Since diagnosis, he had been receiving 8 mg of methotrexate weekly, which maintained his RA disease activity at clinical remission for several years. He also had bronchial asthma diagnosed 5 years earlier, for which he had been prescribed inhaled budesonide, and ossification of the posterior longitudinal ligament.

The patient presented with painful swelling and flexion disorder of the metacarpophalangeal (MP) joint of the right index finger 6 months before his current admission. The swelling had not responded to a single intra-articular injection of 4 mg of triamcinolone acetonide or oral cefalexin (1500 mg daily for 14 days). Painful swelling of the MP joint progressed to involve the entire right index finger 4 weeks before the admission. Since the swelling deteriorated day by day, he was admitted to our hospital for further investigation and treatment.

He regularly engaged in gardening as a hobby and frequently handled soil with his bare hands. He did not recall any specific trauma to the affected finger during gardening activities.

**Figure 1:**
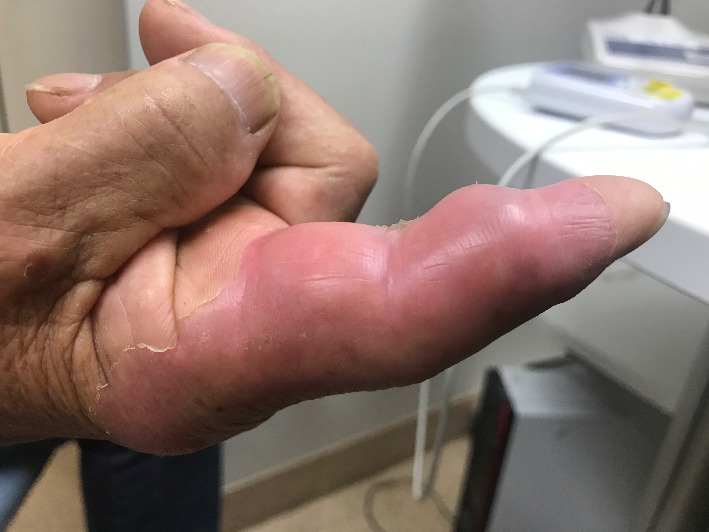
Gross appearance of the lesion in the right index finger. Painful swelling of all soft tissues around the tendon was observed.

His height was 164 cm, weight 64.5 kg, body temperature 37.3°C, heart rate 71 regular beats per minute, blood pressure 137/81 mmHg, and oxygen saturation was 95%. Physical examination yielded no remarkable findings, with the exception of painful swelling of the right index finger (Figure 1).

**Figure 2:**
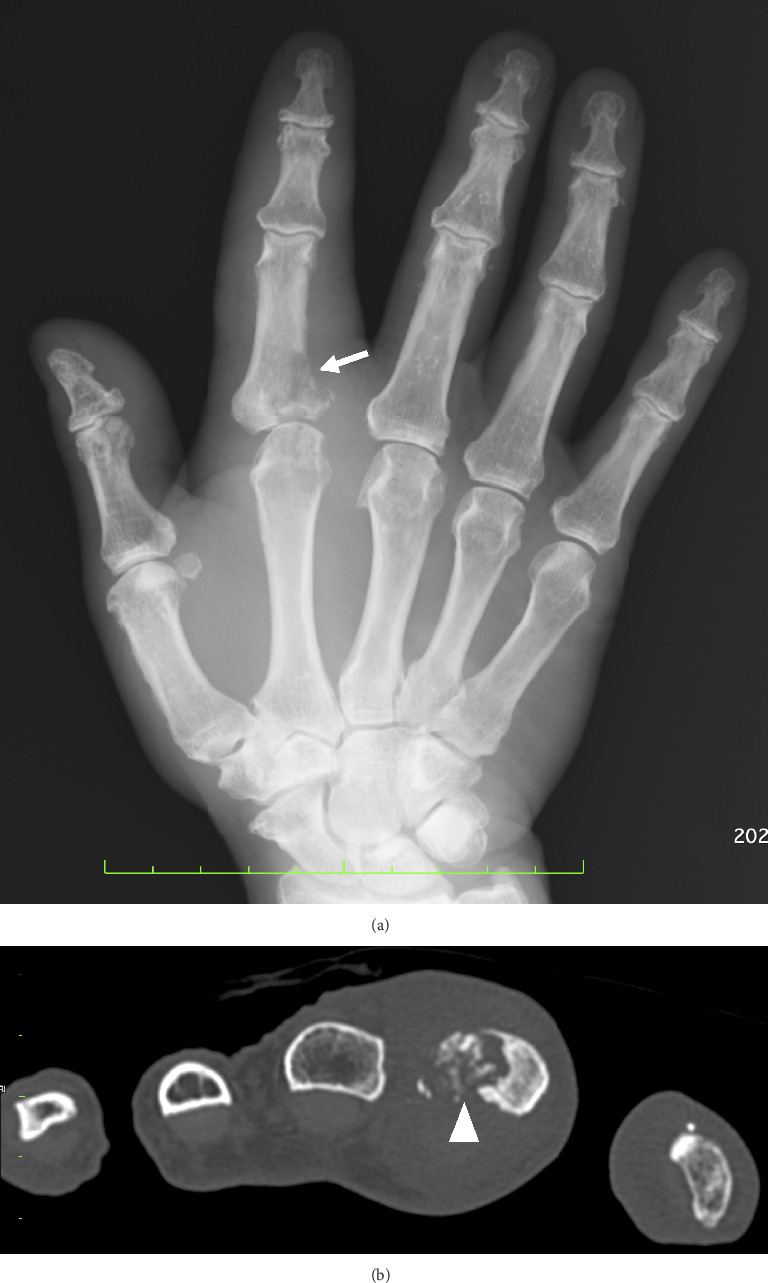
(a) Roentgenogram of the right hand. Bony destruction of the ulnar aspect of the base of the proximal phalanx of the right index finger was observed (arrow). (b) Computed tomography of the right hand. Bone destruction of the ulnar aspect of the base of the proximal phalanx of the right index finger was observed (arrowhead).

His white blood cell count was 6400/μL, with 64.3% neutrophils, 27.4% lymphocytes, 6.5% monocytes, 1.6% eosinophils, and 0.2% basophils. Blood chemistry findings were all within normal limits. C-reactive protein was negative. The erythrocyte sedimentation rate was 9 mm/hour. Although he had a positive reaction to the tuberculin skin test, the interferon-gamma release assay was negative, ruling out exposure to *Mycobacterium tuberculosis*. A roentgenogram and computed tomography of the right fingers showed marked bony destruction on the ulnar side of the MP joint of the proximal phalanx of the right index finger (Figure 2). A chest computed tomography scan was performed at the time of admission and showed no pulmonary lesions such as nodules, infiltrates, or bronchiectasis. There was no radiological evidence of pulmonary involvement by M. *intracellulare*.

**Figure 3:**
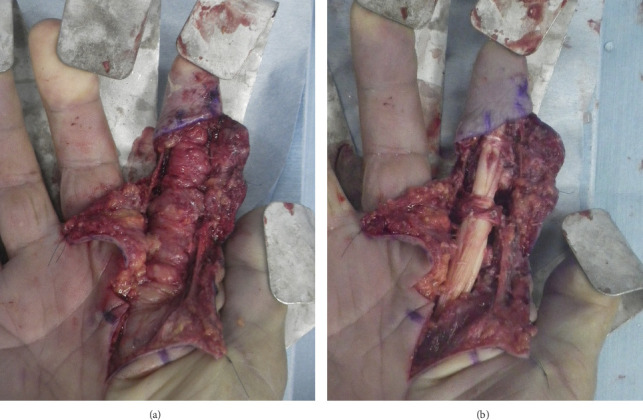
Palmar appearance of the right hand during synovectomy. (a) Infected and thickened tendinous synovium enveloped the entire flexor tendon. (b) After resection of the tendinous synovium.

**Figure 4:**
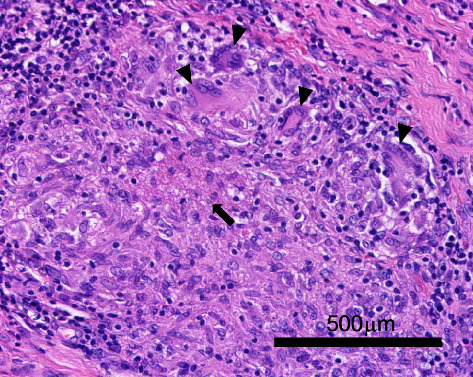
Histopathological findings of the tendinous synovium. Epithelioid cells around multinucleated Langhans giant cells (arrow heads) and caseous necrosis (black arrow) were observed.

Synovectomy was performed for irrigation and resection of the infected tissues. Infectious synovium had involved the flexor tendon, MP joint, and extended into the bone marrow in the base of the proximal phalanx, indicating infiltration into the bone and consistent with osteomyelitis (Figure 3). Microscopically, the infected synovium showed villous growths and included epithelioid granulomas with caseous necrosis (Figure 4). The mycobacterial culture of irrigated synovial fluid became positive for M. *intracellulare* after 28 days of incubation. Drug susceptibility testing revealed that the isolated strain of M. *intracellulare* was sensitive to clarithromycin.

Postoperatively, combination therapy with 750 mg daily of rifampicin, 500 mg daily of ethambutol, and 800 mg daily of clarithromycin was started. After a transient worsening of the swelling 10 days after the induction of antimicrobial treatment, the swelling and pain gradually subsided over approximately 6 weeks. The patient was instructed to perform a range of motion exercises and grip strength training at home. Despite these efforts, significant functional limitations remained, with the MP and PIP joints having only about 10 degrees of flexion.

At the 6-month follow-up, the patient reported satisfaction with the outcome as he was pain-free despite the limited range of motion. The antimicrobial therapy was continued for over 2 years without any significant adverse effects, and there was no recurrence of infection during this period.

## 3. Discussion

Our patient developed chronic tenosynovitis with terrible destruction of the MP joint in the index finger, during the clinical course of well-controlled RA, which deteriorated despite intra-articular injection of triamcinolone acetonide. M. *intracellulare* was detected by mycobacterial culture of irrigated fluid from the synovectomy wound. Epithelioid granulomas with caseous necrosis were found in the resected synovium.

MAC infection occurs in four clinical settings. First, it is seen as a late opportunistic infection in patients with human immunodeficiency virus and is described as the most common cause of systemic MAC infection in these patients [5–7]. Second, pulmonary disease due to MAC is well recognized. Most cases occur in association with preexisting pulmonary diseases associated with dead space in the lungs, such as chronic obstructive pulmonary disease or old cavitary lesions due to preceding *tuberculosis* [8]. Third, disseminated MAC disease has also been described in immunocompromised patients without AIDS. The common predisposing factors are underlying malignancy, collagen vascular disease, and use of glucocorticoids, tumor necrosis factor–alpha inhibitors (TNFis), and cytotoxic agents [9–14]. Fourth, focal extrapulmonary infections, such as isolated urinary tract infection, tenosynovitis, osteomyelitis, and septic arthritis, have been described in several reports in the literature [15–21]. Our case is classified as a localized extrapulmonary infection.

*Mycobacterium intracellulare* infection in patients with RA is rare. Upon literature review, only five cases have been previously reported [9–12, 15]. All documented cases had received systemic glucocorticoid therapy, and four out of the five patients had been treated with cytotoxic agents. Biological agents were administered in three cases, with TNFi selected in two instances. In two reported cases, surgical intervention was presumed to be the route of M. *intracellulare* entry.

Our patient had received methotrexate as a cytotoxic agent but had not undergone systemic glucocorticoid therapy or TNFα inhibition. In addition, there was no history of trauma or surgical procedures that could account for M. *intracellulare* introduction into the body. However, similar to our case, intra-articular local glucocorticoid injections had been performed in two previously reported cases.

In our case, we hypothesize that the patient developed M. *intracellulare* arthritis of the MP joint of the right index finger, potentially triggered by an unnoticed microtrauma. The local injection of triamcinolone acetonide likely induced severe localized immunosuppression, playing a decisive role in exacerbating the infection. While it is theoretically possible that M. *intracellulare* entered the joint through penetrating trauma during the intra-articular injection, this seems unlikely considering that swelling of the MP joint preceded the glucocorticoid injection, and meticulous disinfection was performed during the procedure.

It is not uncommon for RA patients with otherwise well-controlled disease activity through disease-modifying antirheumatic drugs to experience exacerbation in a limited number of joints. In such cases, local injection of triamcinolone acetonide is frequently selected as a treatment option. However, clinicians should carefully consider the possibility that joint swelling may be caused by infection rather than rheumatoid activity and thoroughly evaluate the risk–benefit profile before determining the appropriateness of intra-articular injections.

The patient's regular gardening activities without protective gloves likely exposed him to high concentrations of M. *intracellulare*, as these organisms naturally grow in soil and water. This exposure represents a significant risk factor for NTM infection, particularly in patients with compromised immunity.

Microbiological examinations often show false negative results because of difficulty in culture of *M*. *intracellulare* [16, 20]. Even polymerase chain reaction tests sometimes show false negative results. In patients who develop such infections as a complication, it might be difficult to distinguish the infection from monoarthritis due to a RA flareup in the early stages of the infection.

Cases such as ours should be publicized widely as a rare infectious complication in relation to intra-articular injection of glucocorticoids, and MAC infection should be considered in the differential diagnosis of monoarthritis due to the basic disease in patients with RA-associated musculoskeletal disease.

## Data Availability

All relevant data are available on request due to privacy/ethical restrictions.
